# Cell Polarity Protein Pals1-Associated Tight Junction Expression Is a Favorable Prognostic Marker in Clear Cell Renal Cell Carcinoma

**DOI:** 10.3389/fgene.2020.00931

**Published:** 2020-08-28

**Authors:** Pingping Li, Ping Lan, Sheng Liu, Yaochun Wang, Peijun Liu

**Affiliations:** ^1^ Center for Translational Medicine, The First Affiliated Hospital of Xi’an Jiaotong University, Xi’an, China; ^2^ The Key Laboratory for Tumor Precision Medicine of Shaanxi Province, The First Affiliated Hospital of Xi’an Jiaotong University, Xi’an, China; ^3^ Department of Nephrology, Kidney Hospital, The First Affiliated Hospital of Xi'an Jiaotong University, Xi’an, China; ^4^ Department of Urology, The First Affiliated Hospital of Xi’an Jiaotong University, Xi’an, China

**Keywords:** cell polarity protein Pals1-associated tight junction, clear cell renal cell carcinoma, prognosis, tumor cell proliferation, mitogen-activated protein kinases

## Abstract

**Introduction**: The Pals1-associated tight junction (PATJ) is a Crumbs (CRB) complex component that regulates epithelial cell apico-basal polarity and directional migration. This study assessed PATJ expression in clear cell renal cell carcinoma (ccRCC) vs. normal tissues and associated with ccRCC progression and prognosis.

**Methods**: The effects of PATJ knockdown were investigated on regulation of normal kidney epithelial cell viability and protein expression *in vitro*. The *PATJ* mRNA data in ccRCC were obtained from The Cancer Genome Atlas (TCGA) and Gene Expression Omnibus (GEO) databases and analyzed with UALCAN, LinkedOmics, Kaplan-Meier Plotter, GEPIA, and SurvExpress tools. Immunohistochemistry was performed for PATJ in tissue microarray sections (*n* = 150 ccRCC and 30 normal renal specimens). Normal human kidney tubular epithelial cell (HKC) cells were transfected with *PATJ* and negative control siRNA for cell viability CCK-8 assay, flow cytometry, and western blots.

**Results**: The data showed that *PATJ* mRNA and protein were downregulated in ccRCC tissues and cell lines. Downregulation of *PATJ* mRNA was associated with male patients, advanced tumor stages, grades, and ccB subtypes as well as poorer overall and disease-free survival of patients. Furthermore, PATJ protein was also significantly downregulated in ccRCC tissues and associated with advanced tumor pathologic, TNM stages and poorer overall. *In vitro*, knockdown of PATJ expression promoted HKC proliferation and the activation of mitogen-activated protein kinases (MAPK) pathway proteins.

**Conclusions**: This study revealed that a decrease of PATJ in ccRCC, which was associated with male patients, advanced tumor, and poorer survival, suggesting that PATJ may be a useful prognostic biomarker and therapeutic target for ccRCC.

## Introduction

Renal cell carcinoma (RCC) is a frequently diagnosed kidney cancer in men 65 years or older and clear cell RCC (ccRCC) is the most common histologic subtype (Cohen and McGovern, 2005; Hsieh et al., 2017), counting for approximately 90% of kidney cancers and up to 4% of all newly diagnosed cancer cases in 2018 worldwide (Siegel et al., 2018). Clinically, RCC is usually symptomless and at advanced stages, patients have the classic triad of hematuria, flank pain, and flank mass (Cohen and McGovern, 2005; Hsieh et al., 2017). Approximately 25–30% of RCC patients had metastatic renal cell carcinoma (mRCC) at the time of tumor diagnosis (Lam et al., 2005a), and 70–80% patients are treated with nephrectomy for curative intent or tumor removal. Indeed, for patients with a localized disease within the kidney, surgery alone may be sufficient to cure and the recurrence rate is usually less than 25%. However, patients with advanced tumor progression (tumor has spread outside of the kidney or the locoregional disease) have a great risk of recurrence (more than 40%) after initial treatment (Lam et al., 2005b; Gupta et al., 2008), leading to a 12% of the 5-year survival rate (Joosten et al., 2017). RCC is usually resistant to radiation and chemotherapy (Campbell et al., 2016), as well as to recent treatment options include immunomodulatory and targeted therapies. For example, a combination approach to target tumor angiogenesis using sunitinib, bevacizumab, or pazopanib and the mammalian target of rapamycin (mTOR) pathway using temsirolimus has been approved as a front-line treatment option for mRCC, whereas PD-1/PD-L1 is a more recently developed anti-RCC agent (Barata and Rini, 2017; Posadas et al., 2017; Rodriguez-Vida et al., 2017; Zarrabi and Wu, 2018). However, treatment effectiveness, treatment-related toxicities, drug resistance, and financial burden are all major concerns for RCC patients (Hsieh et al., 2017). Thus, the identification and evaluation of tumor markers for preliminary screening, early metastasis detection, and monitoring of treatment responses and prognosis are sorely needed.

Toward this end, a better understanding of RCC pathogenesis and tumor development and progression can help us effectively control RCC initiation and metastasis. ccRCC originates from the proximal renal tubular epithelium. Cell polarity is a key feature of the epithelium (Halaoui and McCaffrey, 2015), which is maintained through the tight junctions, desmosomes, and adherens junctions (Anderson and Van Itallie, 2009). Molecularly, cell polarity is maintained through the expression of specific proteins in specific areas of the cell membrane. Crumbs (CRB), PAR, and SCRIB polarity complexes specifically regulate the apical and basolateral membrane domains *via* the organization of intracellular signaling pathways for epithelial polarity (Halaoui and McCaffrey, 2015). Dysregulation of these cell polarity proteins could rewire oncogenic and tumor suppressor signaling pathways and promote cell proliferation but inhibit apoptosis and induce tumor cell invasion, metastasis, and differentiation (Halaoui and McCaffrey, 2015; Li et al., 2017). Recent studies have revealed that Pals1-associated tight junctions [PATJ; also called InaD-like protein (INADL)] is a CRB complex component and regulates epithelial cell apico-basal polarity and directional migration by regulating the localization of atypical protein kinase C (aPKC) and PAR3 to the leading edge in Madin-Darby canine kidney II cells (Shin et al., 2007), while the directional migration of blood capillaries during embryonic development was regulated by *patj*/*amot/syx* signaling to control local GTPase activity in zebrafish (Ernkvist et al., 2009).

In this study, we analyzed expression of *PATJ* mRNA using The Cancer Genome Atlas (TCGA) and GEO databases and PATJ protein expression in our retrospective cohort of 150 ccRCC tissues vs. 30 normal renal specimens. We then evaluated the association of PATJ expression with the clinicopathological features and survival of ccRCC patients. After that, we investigated the effect of PATJ expression on the regulation and proliferation of human renal tubular epithelial cells (HKC). We show the utility of PATJ as a diagnostic and prognostic biomarker for ccRCC.

## Materials and Methods

### Retrieval and Analysis of TCGA Database Data

We utilized a web tool (the UALCAN at http://ualcan.path.uab.edu/index.html) to retrieve and analyze tumor transcriptome data on changes in *PATJ* mRNA and protein levels in ccRCC tissues in TCGA database and Clinical Proteomic Tumor Analysis Consortium (CPTAC) Confirmatory/Discovery dataset. This web tool provided us (1) publicly accessible cancer transcriptome data (like TCGA) and (2) The graphs and plots depicting gene expression and patients’ survival data (Chandrashekar et al., 2017). We retrieved data on level of *PATJ* mRNA and protein in ccRCC vs. normal tissues and ccRCC pathological features (gender, stages, grades, and subtypes) from patients from TCGA and CPTAC, and then analyzed using the UALCAN web tool.

### Cell Culture and *PATJ* siRNA Transfection

Human renal tubular epithelial cell line HKC and human ccRCC cell lines Caki-1, Caki-2, and 786-O were obtained from the National Infrastructure of Cell Line Resource (Beijing, China). HKC cells were cultured in Dulbecco’s modified Eagle’s medium (DMEM)/F12 at 1:1 (Coring, Corning, NY, USA) supplemented with NEAA (Gibco, Carlsbad, CA) and 5% fetal bovine serum (FBS; Hyclone, Logan, UT, USA). Caki-1 and Caki-2 cells were grown in the McCoy’5A Medium from Hyclone and supplemented with 10% FBS (Hyclone), while 786-O cells were cultured in Roswell Park Memorial Institute medium-1640 (RPMI1640; Coring) and supplemented with 10% FBS (Hyclone). All cell lines were maintained in a humidified incubator containing 5% CO_2_ at 37°C.

Two *PATJ* siRNA constructs were used to knockdown PATJ expression in cells *in vitro* and their sequences were *PATJ*-1, 5′-GGA UGU CAA UAC UGA AGA ATT-3′ and 5′-UUC UUC AGU AUU GAC AUC CTT-3′; *PATJ*-2, 5′-GCA GAU GGU GUA GCA GAA ATT-3′ and 5′-UUU CUG CUA CAC CAU CUG CTT-3′. These *PATJ* siRNA constructs and a negative control siRNA were purchased from GenePharma Company (Shanghai, China) and used to transiently transfect into HKC cells in 6-cm plates for 48 h [the transfection conditions were 166 pmol siRNA and 17 μl Lipofectamine™ 2000 (Invitrogen, Carlsbad, CA, USA) in 250 μl of Opti-MEM medium (Invitrogen)].

### Quantitative Reverse Transcriptase-Polymerase Chain Reaction

Total cellular RNA was isolated using the RNA Fast 200 (Cat. #220010; Fastagen Biotech, Shanghai, China) and reversely transcribed into cDNA using the PrimeScript™ RT Master Mix (cat. #RR036A, TaKaRa Biotechnology (Dalian) Co., Ltd., China) according to the manufacturers’ protocols. PCR was performed using the TB Green™ Premix Ex Taq™ II (Cat. #RR820A, TaKaRa) in Bio-Rad CFX96 system (Hercules, CA, USA) according to the manufacturer’s instructions. The primers used were *PATJ*, 5′-AAG GGT GAC ACG TCG CAG AA-3′ and 5′-GGC TGA ACA ATC TGA GGG TAT ATG G-3′; *β-actin*, 5′-CAT GTA CGT TGC TAT CCA GGC-3′ and 5′-CTC CTT AAT GTC ACG CAC GAT-3′. The experiment was performed in triplicate and level of *PATJ* mRNA was normalized to β-actin.

### Western Blot

The whole cell lysates were prepared using a modified radioimmunoprecipitation assay (RIPA) buffer and quantified using the Bradford Protein Assay (Bio-Rad). After that, these protein samples were separated in sodium dodecyl sulfate-polyacrylamide gel electrophoresis (SDS-PAGE) gels and transferred onto the polyvinylidene fluoride membranes (PVDF; Millipore, Billerica, MA, USA) for a standard western blot protocol according to a previous study (Kim, 2017). The primary antibodies used were an anti-PATJ antibody (A12063; 1:500; ABclonal, Wuhan, China), the MAPK Family Antibody Sampler Kit (#9926; 1:1000; Cell Signaling Technology, Danvers, MA, USA), Phospho-MAPK Family Antibody Sampler Kit (#9910; 1:1000; Cell Signaling Technology), or anti-β-actin antibody (#AC026; 1:10000; ABclonal), while the secondary antibody was the horseradish peroxidase (HRP)-conjugated IgG from Cell Signaling Technology, the positive protein signals were visualized by using the enhanced chemiluminescence (ECL) Plus kit (Millipore).

### LinkedOmics

We also utilized a web tool (the LinkedOmics at http://www.linkedomics.org/login.php) to analyze the multi-omics data from TCGA database. We were able to identify and analyze mRNA signatures, biomarkers of clinical attributes, and putative target genes of the transcriptional factors, microRNAs, or protein kinases using this web tool. The analytical results were exported as graphic plots (Vasaikar et al., 2018). Thus, we obtained the levels of *PATJ* mRNA associated with ccRCC TNM stages.

### Kaplan-Meier Plotter

After that, we used another web tool known as the Kaplan-Meier Plotter at http://kmplot.com/analysis/ to assess the association of *PATJ* mRNA with overall survival (OS) of ccRCC patients. This web tool contains expression data of 54,675 genes in 18,674 cancer samples for examining their potential association with the prognosis of patients with 5,143 breast, 1,816 ovarian, 2,437 lung, 364 liver, and 1,065 gastric cancers. Moreover, the web tool also contains the miRNA subsystems for an additional 11,456 samples of 20 different cancer types. This web tool enables us to perform a meta-analysis of different genes for biomarker assessment (Lánczky et al., 2016).

### GEPIA

GEPIA at http://gepia.cancer-pku.cn/ is an interactive web tool for us to analyze data on the RNA sequencing expression of 9,736 tumors vs. 8,587 normal samples from TCGA and the GTEx projects and this web tool uses a standard data processing pipeline to provide customizable function analyses, like differential expression of a given gene in tumor vs. normal tissues or profiling of differentially expressed genes according to cancer types or pathological stages, or survival data, etc., which was developed by Dr. Zefang Tang, Dr. Chenwei Li, and Dr. Boxi Kang in Dr. Zhang’s Lab in Peking University (Tang et al., 2017). We utilized this to associate *PATJ* mRNA levels with disease free survival (DFS) of ccRCC patients.

### SurvExpress

The SurvExpress at http://bioinformatica.mty.itesm.mx:8080/Biomatec/SurvivaX.jsp is a versatile online biomarker validation tool to assess the significance of gene expression in prognosis of various cancers (Aguirre-Gamboa et al., 2013). The data on renal clear cell carcinoma tissues were from TCGA (*n* = 468) and in this study, we validated *PATJ* mRNA as a prognostic marker using this web tool.

### Tissue Microarrays and Immunohistochemistry

This study was approved by the Local Ethics Committee of Human Research at the First Affiliated Hospital, Xi'an Jiaotong University (Shaanxi, China). Tissue Microarray (TMA) sections were obtained from Shanghai Outdo Biotech Co., Ltd. (Cat. #HkidE180Su02; Shanghai, China) and contained 150 cases of ccRCC tissues and 30 normal renal specimens. We retrospectively retrieved the medical records and reviewed and analyzed clinicopathological data vs. PATJ expression (Table 1).

**Table 1:** Association of PATJ expression with clinicopathological parameters from ccRCC patients.

		PATJ level		
Variables	No.	− (*n* = 80)	+ (*n* = 70)	*p*
Histologic grade				
1–2	103	50 (48.5%)	53 (51.5%)	<0.0001
3–4	47	30 (63.8%)	17 (36.2%)	
TNM stage				
I	122	60 (49.2%)	62 (50.8%)	0.037
II–IV	28	20 (71.4%)	8 (28.6%)	

For immunohistochemistry, we stained PATJ protein using the TMA sections using an immunohistochemical staining kit (PV-6001; Beijing Zhongshan Golden Bridge Biotechnology Co., Ltd., Beijing, China) according to the manufacturer’s recommended protocol. After deparaffinization and rehydration, TMA sections in the HIER solution (pH 6) were placed into a microwave oven and heated at 100 W for 6 min and 50 W for 13 min for antigen recapture, and then incubated in 3% H_2_O_2_ at room temperature for 10 min for inactivation of potential endogenous peroxidase activity. The sections were then incubated in a blocking solution containing a normal goat serum (1:4) for 15 min and in a primary anti-PATJ antibody (Cat. #HPA066352; Sigma-Aldrich, St Louis, MO, USA; 1:100) at 4°C overnight. On the next day, the sections were washed with PBS thrice, and then further incubated with a secondary antibody at the room temperature for 30 min and subsequently with the ABC reagent in the dark for 30 min. After that, the TMA sections were subjected to a brief color reaction using the 3,3′-diami-nobenzidine (DAB; Zhongshan Golden Bridge Biotechnology Co., Ltd., Beijing, China), and then counterstaining with hematoxylin. The immunostained TMA sections were finally reviewed and scored under a Leica microscope (Model #SCN 400; Mannheim, Germany) by an experienced pathologist in a blind fashion to the patients’ clinical data.

The immunostaining score system was developed according to the staining intensity (0, negative; 1, weakly positive; 2, moderately positive; and 3, strongly positive) and the percentage of stained cells per field (0, no staining; 1, 1–25% of tumor cell stained; 2, 26–50%; 3, 51–75%; and 4, 76–100%). The sum score of the expression level of 0–2 was considered a negative case, whereas the score of 3–12 was considered as a positive case.

### Cell Viability Assay

Cells were seeded into 96-well plates at a density of 10^4^ cells/well and transiently transfected with *PATJ* siRNA constructs and a negative control siRNA for up to 48 h. At different periods of time, 10 μl of the CCK-8 reagent was added (Saint-Bio, Shanghai, China) and cells were further incubated at 37°C for 3 h and the optical density (OD) values were then measured using a microplate reader (PerkinElmer, Waltham, MA, USA) at the wavelength of 490 nm. Each experiment was in triplicate and repeated at least three times.

### Fluorescence-Activated Cell Sorting

To assess the effects of *PATJ* siRNA, we sorted cells after gene transfection using Fluorescent-activated cell sorting (FACS) and the PI and carboxyfluorescein diacetate succinimidyl ester (CFSE) staining. Briefly, cells were grown and transiently transfected with *PATJ* and negative control siRNA (see above for the details) for 48 h, and then stained with 0.5 mL PI/RNase Staining Buffer (#550825, BD, San Jose, CA) for 15 min at room temperature or 5 μM CFSE for 15 min at 37°C. The cells were then sorted by using FACS (BD).

### Statistical Analysis

All statistical analyses were performed using GraphPad Prism Version 7.0 (GraphPad Software, La Jolla, CA, USA), i.e., to compare the differences between two groups, we carried out the paired *t* test or Mann-Whitney test, while to compare the differences among multiple groups, we performed the one-way analysis of variance (ANOVA) followed by using Dunnett’s multiple comparisons test. The association between PATJ expression and clinicopathological characteristics was assessed using the chi-square test or Fisher’s exact test. All statistical tests were assessed for the two-sided and a value of *p* < 0.05 was considered as statistically significant.

## Results

### Downregulation of *PATJ* mRNA and Protein in ccRCC Tissues and Cell Lines

**Figure 1:**
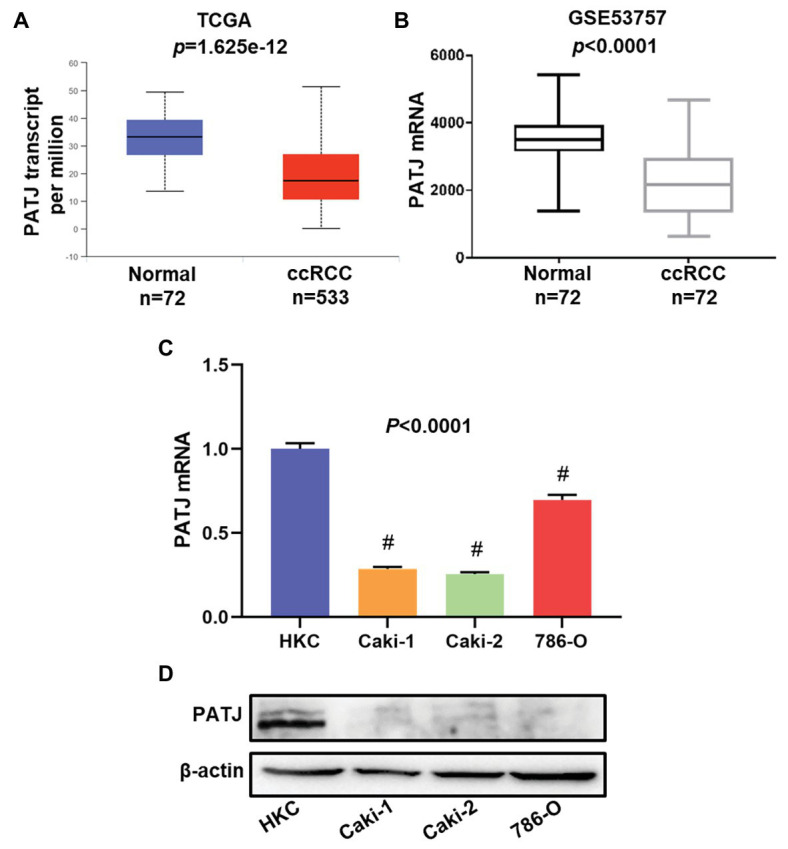
Downregulation of *PATJ* mRNA in clear cell renal cell carcinoma (ccRCC) tissues and cell lines. **(A)** The Cancer Genome Atlas (TCGA) database data. The box plots and *p* were generated using The UALCAN (http://ualcan.path.uab.edu/index.html). **(B)** GSE53757 data on *PATJ* mRNA in 72 ccRCC patients. **(C)** Expression analysis of *PATJ* mRNA in a human renal tubular epithelial cell HKC line and three ccRCC cell lines detected by using quantitative reverse transcriptase-polymerase chain reaction (qRT-PCR). ^#^ *p* < 0.01 and compare to (HKC) cell. **(D)** Western blot. A HKC cell line and three ccRCC cell lines were grown and subjected to Western blot analysis of PATJ protein.

In this study, we first retrieved *PATJ* data on ccRCC vs. normal tissues from the TCGA database and analyzed their expression using the UALCAN web tool. We revealed 533 cases of ccRCC and 72 normal kidney tissues and found that *PATJ* mRNA levels were significantly (*p* = 1.625e-12) reduced in ccRCC tissues compared to normal tissues (Figure 1A). After that, we performed a bioinformatic analysis of *PATJ* expression in 72 ccRCC patients using the GSE53757 data set and revealed a significant decrease in *PATJ* mRNA levels in ccRCC tissues compared with matched normal tissues (*p* < 0.0001; Figure 1B).

Moreover, we determined the level of *PATJ* mRNA and protein in normal human renal tubular epithelial HKC cell vs. ccRCC cell lines using quantitative reverse transcriptase-polymerase chain reaction (qRT-PCR) and western blots. Our data showed that HKC cells expressed the highest level of *PATJ* mRNA and protein among these cell lines, whereas the level of PATJ was much lower in all ccRCC cell lines (Figures 1C,D). These data indicate that the expression of *PATJ* mRNA and protein was downregulated in different sets of ccRCC samples (both TCGA and GEO databases) and cell lines.

### Association of *PATJ* mRNA and Pathological Features in ccRCC

**Figure 2:**
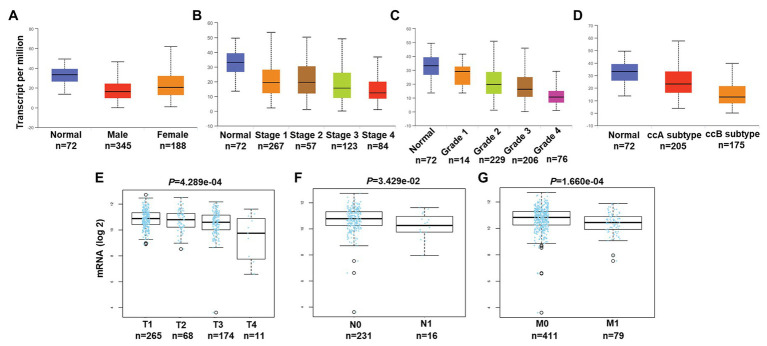
Association of *PATJ* mRNA level with pathological features from ccRCC patients. **(A)** Association of *PATJ* mRNA with patients’ gender. **(B)** *PATJ* mRNA with ccRCC stages. **(C)** *PATJ* mRNA with ccRCC grades. **(D)** *PATJ* mRNA with ccRCC subtypes. The box plots and *p* (Table 2) in **A–D** were generated by using the UALCAN (http://ualcan.path.uab.edu/index.html). **(E)** *PATJ* mRNA with ccRCC T stage. **(F)** *PATJ* mRNA with ccRCC N stage. **(G)** *PATJ* mRNA with ccRCC M stage. The box plots and values *p* (Table 2) in **E–G** were obtained from the LinkedOmics (http://www.linkedomics.org/login.php).

We then associated levels of *PATJ* mRNA with pathological features from ccRCC patients using the UALCAN web tool. The results showed that the decrease in *PATJ* mRNA is associated with males, advanced tumor stages, grades, and ccB subtypes (Figures 2A–D) and the values of *p* are shown in Table 2.

**Table 2:** Association of *PATJ* mRNA and ccRCC pathological features.

Comparison groups	*p*
Normal-vs.-Male	<1E-12
Normal-vs.-Female	2.169E-07
Male-vs.-Female	2.101E-07
Normal-vs.-Stage 1	4.908E-12
Normal-vs.-Stage 2	1.921E-05
Normal-vs.-Stage 3	1.776E-15
Normal-vs.-Stage 4	1.625E-12
Stage 1-vs.-Stage 3	5.997E-03
Stage 1-vs.-Stage 4	1.336E-06
Stage 2-vs.-Stage 4	2.839E-03
Normal-vs.-Grade 2	8.160E-11
Normal-vs.-Grade 3	1.625E-12
Normal-vs.-Grade 4	1.624E-12
Grade 1-vs.-Grade 2	3.920E-02
Grade 1-vs.-Grade 3	3.003E-03
Grade 1-vs.-Grade 4	1.799E-04
Grade 2-vs.-Grade 3	1.664E-02
Grade 2-vs.-Grade 4	1.657E-12
Grade 3-vs.-Grade 4	2.960E-08
Normal-vs.-ccA subtype	5.605E-05
Normal-vs.-ccB subtype	1.624E-12
ccA subtype-vs.-ccB subtype	1.624E-12

The value of *p* was generated by using the UALCAN (http://ualcan.path.uab.edu/index.html).

Moreover, we also analyzed the association between level of *PATJ* mRNA and pathological features from ccRCC patients using the LinkedOmics web tool. Our results further indicated that *PATJ* mRNA was correlated with advanced T stage (*p* = 4.289e-04; *n* = 533; Figure 2E), N stage (*p* = 3.429e-04; *n* = 256; Figure 2F), and M stage (*p* = 1.660e-04; *n* = 501; Figure 2G). These results indicate that decreases in *PATJ* mRNA were related to ccRCC progression.

### *PATJ* mRNA as an Independent Favorable Prognostic Factor for ccRCC

**Figure 3:**
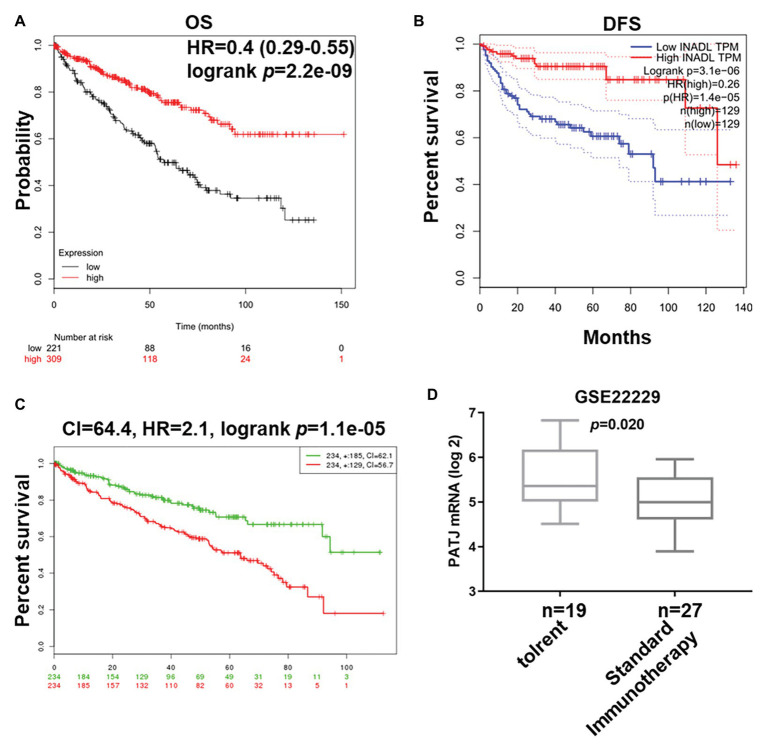
Association of *PATJ* mRNA levels with ccRCC prognosis. **(A)** Kaplan-Meier curves of 530 ccRCC patients stratified by *PATJ* mRNA level analyzed by using Kaplan-Meier Plotter (http://kmplot.com/analysis/). **(B)** Kaplan-Meier curves of 258 ccRCC patients stratified by *PATJ* mRNA level analyzed by using the GEPIA (http://gepia.cancer-pku.cn/). **(C)** Kaplan-Meier curves of 468 ccRCC patients stratified by *PATJ* mRNA level analyzed by using the SurvExpress (http://bioinformatica.mty.itesm.mx:8080/Biomatec/SurvivaX.jsp). **(D)** *PATJ* expression data from GSE22229 were stratified by patients with kidney transplantation.

We next associated *PATJ* mRNA levels with survival of ccRCC patients using Kaplan-Meier curves and the log-rank test in the Kaplan-Meier Plotter^1^. We found that ccRCC patients with low *PATJ* mRNA expression had a significantly shorter OS than patients with high *PATJ* mRNA expression [*p* = 2.2e-09; HR = 0.4 (0.29–0.55); Figure 3A]. We analyzed *PATJ* mRNA expression vs. disease-free survival (DFS) of ccRCC patients using the GEPIA^2^. Our data showed that patients with low *PATJ* mRNA expression had a significantly poorer DFS than patients with high expression (*p* = 3.1e-06; HR = 0.26; Figure 3B). In addition, the SurExpress database data showed that levels of *PATJ* was low in high-risk ccRCC patients using COX regression analysis (*n* = 468; *p* = 1.1e-05; CI = 64.4; HR = 2.1; Figure 3C), while a bioinformatic analysis of *PATJ* expression in 46 patients with kidney transplants using the GSE22229 data set showed a significant decrease in *PATJ* mRNA in standard immunotherapeutic tissues compared with tolerant tissues (*p* = 0.020; Figure 3D), indicating that patients with high *PATJ* expression were more tolerant to kidney transplantation.

### Expression and Association of PATJ Protein in ccRCC vs. Adjacent Normal Renal Tissues With Patients’ Pathological Data

**Figure 4:**
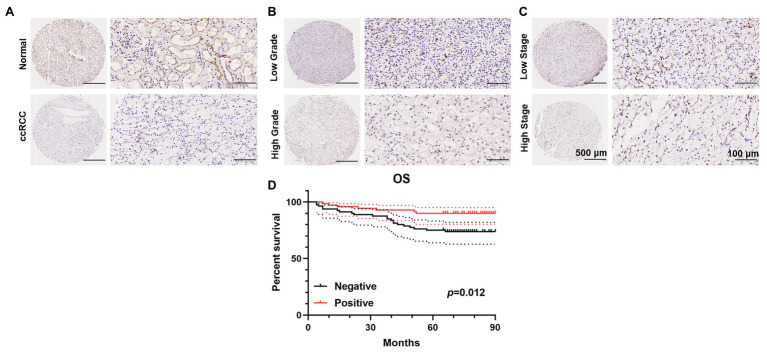
Immunohistochemical detection of PATJ protein in 150 ccRCC vs. normal tissues. The TMAs containing 150 ccRCC and 30 normal renal tissue specimens were immunostained with an anti-PATJ antibody and quantified. Representative fields of view (FOV) show the TMA cores of PATJ expression. **(A)** PATJ expression in normal renal specimens vs. ccRCC. **(B)** PATJ expressions in low grade vs. high grade ccRCC. **(C)** PATJ expressions in low stage vs. high stage ccRCC. **(D)** Patients with negative PATJ expression showed a significantly shorter OS than patients with positive PATJ expression (Log-rank test).

To further validate the decrease in PATJ expression in ccRCC and its association with patients’ pathological data, we performed immunohistochemistry of tissue microarrays containing 150 cases of ccRCC and 30 adjacent normal renal tissues. We found that PATJ protein was predominantly expressed in the membrane of renal tubular epithelial cells, but was reduced in ccRCC tissues (Figure 4A), i.e., 53.3% of ccRCC tissues lost PATJ expression (*p* = 0.027, Fisher’s exact test; Table 3). The loss of PATJ protein was significantly associated with advanced histologic grades and TNM stages (Figures 4B,C and Table 1).

Of these 150 patients, 70 patients showed positive PATJ expressed tumor, whereas 80 patients showed a negative PATJ expressed tumor. Patients with a negative PATJ expressed tumor had a significantly shorter OS than patients with a positive expressed tumor (*p* = 0.012 analyzed by using the log-rank test; Figure 4D). The result indicated that PATJ may be a prognostic indicator for ccRCC patients.

### Expression and Association of PATJ Protein in ccRCC vs. Adjacent Normal Renal Tissues From CPTAC Dataset

**Figure 5:**
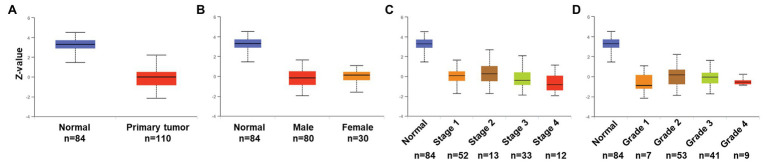
Association of PATJ protein level with pathological features from ccRCC patients. **(A)** Downregulation of PATJ in ccRCC tissues. **(B)** Association of PATJ with patients’ gender. **(C)** PATJ with ccRCC stages. **(D)** PATJ with ccRCC grades. The box plots and values of *p* (Table 3) in **A–D** were generated by using the UALCAN (http://ualcan.path.uab.edu/index.html).

To validate expression and association of PATJ protein in ccRCC with patients’ pathological data, we utilized the UALCAN web tool and found that PATJ protein was reduced in ccRCC tissues vs. normal renal tissues (Figure 5A) and decrease in PATJ protein was associated with advanced tumor stages (Figure 5C; Table 4).

**Table 3:** Association of PATJ protein and ccRCC pathological features.

Comparison groups	*p*
Normal-vs.-Primary	1.765E-53
Normal-vs.-Male	1.253E-48
Normal-vs.-Female	3.659E-17
Normal-vs.-Stage 1	2.116E-34
Normal-vs.-Stage 2	1.003E-06
Normal-vs.-Stage 3	4.255E-23
Normal-vs.-Stage 4	1.258E-09
Stage 1-vs.-Stage 4	2.071E-02
Stage 2-vs.-Stage 4	3.490E-02
Normal-vs.-Grade 1	7.409E-05
Normal-vs.-Grade 2	2.121E-34
Normal-vs.-Grade 3	5.408E-26
Normal-vs.-Grade 4	4.228E-07

The value of *p* were generated by using the UALCAN (http://ualcan.path.uab.edu/index.html).

**Table 4:** Differential expression of PATJ protein between ccRCC and adjacent normal kidney tissues.

		PATJ level		
Variables	*n*	− (*n* = 89)	+ (*n* = 91)	*p*
Normal	30	9 (30%)	21 (70%)	0.027
ccRCC	150	80 (53.3%)	70 (46.7%)	

### Promotion of HKC Cell Proliferation After Knockdown of PATJ Expression *in vitro*

**Figure 6:**
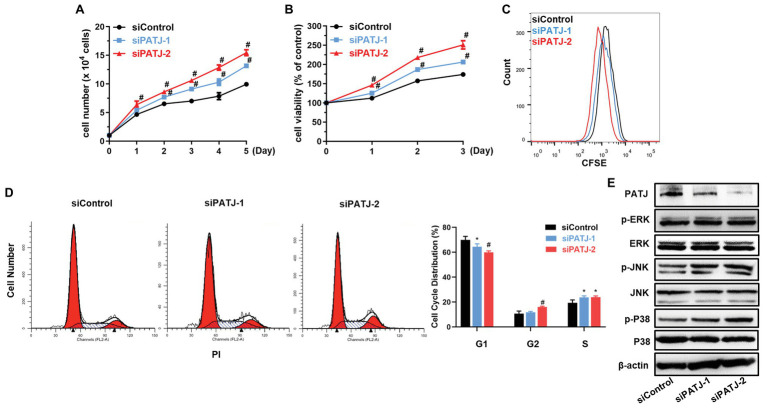
Promotion of human renal tubular epithelial cell HKC proliferation after knockdown of PATJ protein. **(A)** Growth curves of siPATJ transfected HKCs. **(B)** Cell viability using the CCK-8 assay. The percentage of cell viability was calculated from the OD values of the test groups normalized to the control group. **(C)** The CFSE and FACS assay. **(D)** Flow cytometric analysis of cell cycle. **(E)** Western blot. ^*^ *p* < 0.05; and ^#^ *p* < 0.01.

Next, we further investigated the effect of PATJ knockdown on the regulation of human renal tubular epithelial cell viability. As shown in Figure 6A, growth of PATJ-downregulated KHC cells was induced compared with that of control cells, which were consistent to cell viability (Figure 6B) and the CFSE and flow cytometry (Figure 6C). PATJ-knockdown HKC cells appeared to mainly accumulate in the S phase of the cell cycle, whereas the number of cells in the G1 phase was decreased significantly (Figure 6D).

After that, we assessed levels of proteins that may contribute to the increase in HKC viability, such as the mitogen-activated protein kinases (MAPK) pathway [the extracellular signal-regulated kinase (ERK), c-Jun N-terminal kinase (JNK), and P38]. We found that levels of phospho-ERK, phospho-JNK, and phospho-P38 were all increased after knockdown of PATJ expression, whereas levels of their total proteins remained unchanged (Figure 6E), indicating that PATJ downregulation potentially promotes HKC cell viability by activation of the MAPK pathway.

## Discussion

Dysregulation of cell polarity and cell polarity protein expression has been well-documented in the literature as a key step in cancer development and progression (Apodaca, 2018). The mammalian CRB polarity complex containing the integral membrane protein CRB3, PATJ, and PALS1 plays a key role in the establishment and maintenance of epithelial apicobasal polarity and tissue morphogenesis (Michgehl et al., 2017). Our previous study revealed that CRB3 was a favorable independent prognostic factor in ccRCC (Mao et al., 2015). In this study, we assessed the expression of PATJ, a CRB component, for association with ccRCC clinicopathological features and prognosis. We found that levels of both *PATJ* mRNA and protein were downregulated in ccRCC tissues and cell lines compared with healthy controls and cell lines. Reduced *PATJ* mRNA was associated with male patients, advanced tumor stages, grades, and ccB subtypes as well as poorer overall and DFS of patients. Our own experimental data showed that PATJ protein was also significantly downregulated in ccRCC vs. normal tissues, which was associated with advanced tumor pathologic stage and TNM stage, and patients with a negative PATJ expressed tumor had a significantly shorter OS. Moreover, knockdown of PATJ expression induced HKC cell proliferation and activation of MAPK pathway proteins. In conclusion, this study demonstrated that PATJ expression was downregulated in ccRCC and reduced PATJ level was associated with male patients and advanced tumor and poorer survival. Future studies will assess PATJ levels as a ccRCC prognostic biomarker and therapeutic target, such as, increase in PATJ expression in ccRCC cell lines by PATJ cDNA.

Indeed, the advantage of the UALCAN web, as an integrated data-mining platform, contains data of the potential cancer transcriptome using the TCGA level 3 RNA-seq and patients’ clinicopathological features on 31 cancer types. This website has been now visited over 300,000 times by cancer researchers from over 100 countries and cited over 500 times (Chandrashekar et al., 2017). However, the UALCAN web tool does not provide the original data for users to further analyze; thus, it is not possible for us to correct any of the *p*-values for the false-discovery rate (FDR).

PATJ protein was originally identified localized in the tight junctions of epithelial cells (Bhat et al., 1999; Céline et al., 2002; Yoko et al., 2002) and was a novel regulatory element of the polycystin-2 (PC2) channel and involved in autosomal dominant polycystic kidney disease (ADPKD) (Duning et al., 2010). A previous study reported a *PATJ* somatic mutation in two of the three PD-L1-positive ccRCC cases (Wang et al., 2018); thus, suggesting that PATJ might be a promising predictive factor for PD-L1 expression in ccRCC cells. However, to date, there is no study reporting PATJ expression in ccRCC, to the best of our knowledge. In the current study, we observed high PATJ expression in kidney epithelial cells but PATJ expression was reduced in ccRCC tissues and cell lines. Previous studies showed that epithelial-mesenchymal transition (EMT) markers SANIL and ZEB1 could transcriptionally inhibit PATJ expression in MDCK and MDA-MB-231 cells (Aigner et al., 2007; Whiteman et al., 2008). *PATJ* SNP variants were associated with worse outcomes in ischemic stroke patients (Mola-Caminal et al., 2019). However, the cause of PATJ downregulation in ccRCC requires future investigation. Indeed, ccRCC frequently occurs in aged male patients (Cohen and McGovern, 2005), indicating that patients may have an aggressive ccRCC, further confirmed in our current study of PATJ expression. It could be obvious that PATJ, a protein at tight junction, does function to maintain and regulates the epithelial cell polarity and decrease in PATJ expression promotes ccRCC progression, which is supported by our current data. However, to date, it is still unknown how PATJ expression is dysregulated in human cancers, including ccRCC and whether restoration of PATJ expression could suppress tumor progression as a novel therapeutic target.

In our current study, we found that PATJ was predominantly expressed in the nuclei of ccRCC cells. A previous study reported by Cho et al. showed PATJ localization in the nuclei of the apically located subset of early embryonic retinal progenitor cells (Kim et al., 2015). Nuclear PATJ localization in subsets of mitotic cells, if proven, might indicate a different function of PATJ protein. As progenitor cells possess stem cell characteristics, it may be that nuclear PATJ localization in progenitor cells may underlie its function in maintaining stemness. Furthermore, our current study revealed that downregulated PATJ expression was associated with poorer overall and DFS of ccRCC patients. These data indicate that PATJ expression could block ccRCC progression and be associated with treatment responses, although future study is needed to confirm this speculation. Indeed, our current data revealed that knockdown of PATJ expression enhanced HKC proliferation and activity of the MAPK pathway proteins *in vitro*, which indirectly supports the role of PATJ in suppression of ccRCC progression. Furthermore, the MAPK pathway contains a cascade of protein kinases including ERK, c-Jun N-terminal kinase (JNK), and p38. The latter is a highly conserved signal transduction in eukaryotic cells and one of the best-characterized signaling cascades to regulate cell proliferation (Achkar et al., 2018; Liu et al., 2018; Liang and Yang, 2019). A number of studies have demonstrated a role for the increased activity of MAPK signaling cascades in RCC cells and activation of the MAPK pathway was generally recognized for RCC growth (Wu et al., 2018; Zhao et al., 2018). Our current data further support this notion.

In conclusion, this study demonstrated a reduction of *PATJ* mRNA and protein in ccRCC tissues and cell lines, downregulation of which was associated with male patients and advanced tumors as well as poorer overall and DFS of patients. Our *in vitro* data further support the role of PATJ in ccRCC progression. Our current finding suggests that detection of PATJ expression may be used as an independent favorable prognostic factor for ccRCC patients and that PATJ should be further evaluated as a novel target for future control of ccRCC.

## Data Availability Statement

All datasets generated for this study are included in the article/supplementary material.

## Ethics Statement

The studies involving human participants were reviewed and approved by the ethics committee of human subject research of the First Affiliated Hospital, Xi’an Jiaotong University (Xi’an, China). The patients/participants provided their written informed consent to participate in this study.

## Author Contributions

PLi analyzed and interpreted the patient data regarding the database. PLa, SL, and YW analyzed the data and given some scientific advices. PLi and PLiu wrote the manuscript, and PLiu supervised all experiments. All authors contributed to the article and approved the submitted version.

### Conflict of Interest

The authors declare that the research was conducted in the absence of any commercial or financial relationships that could be construed as a potential conflict of interest.

## Footnotes

^1^http://kmplot.com/analysis/

^2^http://gepia.cancer-pku.cn/
